# Impact of Unmet Social Needs, Scarcity, and Future Discounting on Adherence to Treatment in Children With Asthma: Protocol for a Prospective Cohort Study

**DOI:** 10.2196/37318

**Published:** 2023-03-07

**Authors:** Olivier Drouin, Tamara Perez, Tracie A Barnett, Francine M Ducharme, Eric Fleegler, Arvin Garg, Kim Lavoie, Patricia Li, Marie-Élaine Métras, Serge Sultan, Sze Man Tse, Jiaying Zhao

**Affiliations:** 1 CHU Sainte-Justine Research Centre Montreal, QC Canada; 2 Division of General Pediatrics Department of Pediatrics CHU Sainte-Justine Montréal, QC Canada; 3 Department of Pediatrics Faculty of Medicine Université de Montréal Montreal, QC Canada; 4 Department of Social and Preventive Medicine School of Public Health Université de Montréal Montreal, QC Canada; 5 Department of Family Medicine Faculty of Medicine McGill University Montreal, QC Canada; 6 Clinical Research and Knowledge Transfer Research Centre Department of Pediatrics CHU Sainte-Justine Montreal, QC Canada; 7 Division of Emergency Medicine Boston Children’s Hospital Boston, MA United States; 8 Department of Pediatrics Harvard Medical School Boston, MA United States; 9 Department of Pediatrics University of Massachusetts Medical School Boston, MA United States; 10 Division of General Academic Pediatrics, Department of Pediatrics Boston Medical Center and School of Medicine Boston University Boston, MA United States; 11 Montreal Behavioural Medicine Centre CIUSSS du Nord-de-l'Ile-de-Montreal Montreal, QC Canada; 12 Department of Psychology Université de Quebec à Montreal Montreal, QC Canada; 13 Centre for Outcomes Research and Evaluation McGill University Health Centre Research Institute Montreal, QC Canada; 14 Department of Pediatrics McGill University Montreal, QC Canada; 15 Division of General Pediatrics Montreal Children's Hospital Montreal, QC Canada; 16 Pharmacy Department and Pharmacy Practice Research Unit CHU Sainte-Justine Montreal, QC Canada; 17 Department of Psychology Faculty of Arts and Sciences Université de Montréal Montreal, QC Canada; 18 Division of Respiratory Medicine, Department of Pediatrics Sainte-Justine University Hospital Center University of Montreal Montreal, QC Canada; 19 Department of Psychology University of British Columbia Vancouver, BC Canada; 20 Institute for Resources, Environment and Sustainability University of British Columbia Vancouver, BC Canada

**Keywords:** asthma, adherence, unmet social needs, behavioral sciences, future discounting, scarcity, pediatrics

## Abstract

**Background:**

Asthma is one of the most prevalent chronic diseases of childhood and disproportionately affects children with lower socioeconomic status. Controller medications such as inhaled corticosteroids significantly reduce asthma exacerbations and improve symptoms. However, a large proportion of children still have poor asthma control, in part owing to suboptimal adherence. Financial barriers contribute to hindering adherence, as do behavioral factors related to low income. For example, unmet social needs for food, lodging, and childcare may create stress and worry in parents, negatively influencing medication adherence. These needs are also cognitively taxing and force families to focus on immediate needs, leading to scarcity and heightening future discounting; thus, there is the tendency to attribute greater value to the present than to the future in making decisions.

**Objective:**

In this project, we will investigate the relationship between unmet social needs, scarcity, and future discounting as well as their predictive power over time on medication adherence in children with asthma.

**Methods:**

This 12-month prospective observational cohort study will recruit 200 families of children aged 2 to 17 years at the Asthma Clinic of the Centre Hospitalier Universitaire Sainte-Justine, a tertiary care pediatric hospital in Montreal, Canada. The primary outcome will be adherence to controller medication, measured using the proportion of prescribed days covered during follow-up. Exploratory outcomes will include health care use. The main independent variables will be unmet social needs, scarcity, and future discounting, measured using validated instruments. These variables will be measured at recruitment as well as at 6- and 12-month follow-ups. Covariates will include sociodemographics, disease and treatment characteristics, and parental stress. Primary analysis will compare adherence to controller medication, measured using the proportion of prescribed days covered, between families with versus those without unmet social needs during the study period using multivariate linear regression.

**Results:**

The research activities of this study began in December 2021. Participant enrollment and data collection began in August 2022 and are expected to continue until September 2024.

**Conclusions:**

This project will allow the documentation of the impact of unmet social needs, scarcity, and future discounting on adherence in children with asthma using robust metrics of adherence and validated measures of scarcity and future discounting. If the relationship between unmet social needs, behavioral factors, and adherence is supported by our findings, this will suggest the potential for novel targets for integrated social care interventions to improve adherence to controller medication and reduce risk across the life course for vulnerable children with asthma.

**Trial Registration:**

ClinicalTrials.gov NCT05278000; https://clinicaltrials.gov/ct2/show/NCT05278000

**International Registered Report Identifier (IRRID):**

PRR1-10.2196/37318

## Introduction

### Background

Asthma is one of the most prevalent chronic diseases of childhood, affecting 1 in 10 children and >3 million Canadians [1,2]. Asthma disproportionately affects children with lower socioeconomic status (SES) [3,4], who not only have a higher prevalence of asthma [5,6] but also have heavier symptom burdens, higher rates of emergency department (ED) visits [7-9], and more hospital admissions [2,4]. The higher burden of disease in these children has several overlapping causes including epigenetic changes related to stress during pregnancy [10], higher secondhand smoke exposure [9,11-13], and greater likelihood of living in environments where air quality is poor [8,14] and allergens are present [15]. Poorer housing quality is also associated with worse asthma control [13,16]. Moreover, families from lower SES have higher perceived barriers to care [17] and are more likely to have unmet care needs [18,19].

Controller medications such as inhaled corticosteroids significantly reduce asthma exacerbations while improving lung function and asthma symptom control [20-22]. Nonetheless, a large proportion of children have poor asthma control, in part owing to suboptimal adherence to inhaled corticosteroids, with objective adherence values ranging from 50% to 70% [23-26]. Previous reports [27-29] suggest that children with lower SES have lower adherence to controller medications, leading to worse asthma control and aggravating health inequities.

Adherence can be hindered by financial barriers that limit access to medication [8,19]. In addition, unmet social needs for food, lodging, and childcare may create stress and worry in parents, negatively influencing medication adherence [30]. Precarious employment, food insecurity, and unstable housing situations are also cognitively taxing and force families to focus on immediate needs [31,32].

Scarcity refers to a state in which one’s cognitive resources such as attention and executive control are taxed by living with insufficient resources (money and time) [31]. Individuals may experience scarcity if they have high levels of stress at work, a relative who is acutely sick, or if they are in an unstable housing situation [32]. Although scarcity can affect anyone, those living in poverty may be particularly susceptible, given the financial constraints that require them to carefully balance immediate needs and upcoming expenses [31]. In pediatric asthma, the apparent unpredictable course of the disease is taxing for families. Psychological distress is frequently reported in qualitative studies of children with asthma and their parents [33], and caregiver life stress is associated with a heavier burden of asthma symptoms [34]. The relationship between stress and adherence in pediatric asthma has shown mixed results [35-37], suggesting that other variables such as scarcity may mediate the interaction.

In addition, poverty forces families to attend to immediate needs and makes it more challenging to think about, and invest in, the distant future. Future discounting represents the relative value one assigns to the future compared with the present [38,39]. Poverty is associated with higher levels of future discounting [40], which has been hypothesized to mediate the impact of lower SES on health outcomes [31,41,42]. Lower levels of future discounting (attributing a greater relative value to the future) are associated with several positive health behaviors, including influenza vaccination [43,44], physical activity [44,45], and abstinence from smoking [44,46-49]. In pediatrics, higher levels of future discounting correlate with lower medication adherence in type 1 diabetes [50,51]. In asthma, as in other pediatric chronic diseases, parents are asked to administer medications to their children daily to prevent future exacerbations and negative health outcomes. Levels of future discounting correlate with adherence to controller medication in young adults with asthma [52], but whether the same is true in children is unknown. Future discounting may be especially important to study in pediatrics, as medication adherence is a shared responsibility between parents and children [53-59] that evolves with the child’s age and developmental stage [53-60]. Children tend to be more present focused than parents [61], which may explain the decreasing levels of adherence to controller medication as the child or adolescent assumes greater responsibility for managing their asthma treatment [62].

In this project, we aim to investigate the relationship between unmet social needs, medication adherence, scarcity, and future discounting in pediatric asthma.

### Scientific Objectives

The overall aims of this project are to determine whether scarcity and future discounting are associated with medication adherence in children with asthma and to explore their role in the relationship between unmet social needs and adherence. Using a prospective observational study, we will investigate three primary relationships:

1. The relationship between scarcity, future discounting, and adherence to asthma controller medication;
2. The association between unmet social needs, scarcity, and future discounting; and
3. The potentially mediating role of scarcity and future discounting on the relationship between unmet social needs and adherence to medication in children with asthma.

Our hypotheses related to each of these three primary relationships are the following:

1. We hypothesize that individuals experiencing scarcity and those with higher rates of future discounting will have lower adherence to asthma controller medication.
2. We hypothesize that unmet social needs will be related to scarcity and future discounting in the following ways:
  - Parents reporting unmet social needs will be more likely to experience scarcity and have greater levels of future discounting;
  - The number of unmet social needs will correlate with level of scarcity and future discounting; and
  - Improvements in a family’s unmet social needs during the study period will be associated with decreased scarcity and future discounting rates.
3. We expect that scarcity and future discounting will play a mediating role in the relationship between unmet social needs and adherence to medication, with higher levels of both scarcity and future discounting specifically amplifying the impact of unmet social needs on medication adherence.

We will also examine two exploratory aims:

1. Examine whether adherence to medication mediates the association between unmet social needs and asthma outcomes: asthma control, ED visits, and hospitalizations.
2. Document the progression of unmet social needs as the world moves to the next phase of the COVID-19 pandemic.

This project will help understand the relationship between cognitive factors, medication adherence, and unmet social needs, opening the door to developing targeted interventions to improve adherence to medication and, ultimately, children’s health outcomes.

## Methods

### Study Design and Setting

This 12-month prospective observational cohort study will recruit 200 families at the Asthma Clinic of the Centre Hospitalier Universitaire Sainte-Justine, a tertiary care pediatric hospital in Montreal, Canada. The asthma clinic has a routine standardized collection of patient demographics (age and sex), disease (phenotype, control, and triggers), and treatment (dose, format, and frequency) characteristics. With patients’ consent, clinical data can be linked with the *Registre de données sur les Medicaments* (reMed) database [63], which collects all data on medication dispensed by all pharmacies in the province of Québec.

### Ethics Approval

The study was reviewed and approved by the Institutional Review Board of the Centre Hospitalier Universitaire Sainte-Justine Research Centre (protocol no: 2022-3888) and has been registered (ClinicalTrials.gov ID: NCT05278000).

### Participants

Parents of children aged 2-17 years seen at the clinic with a diagnosis of asthma, made according to Canadian national guidelines [64], will be eligible for the study. This age range will help us explore whether the relationships identified vary by the age group of the child (preschool vs school-age vs adolescents).

The inclusion criteria will include (1) children with documented prescription for a controller medication at recruitment visit including at least one of the following: inhaled corticosteroids, long-acting beta-agonists, or leukotriene receptor antagonists and (2) consent to access the reMed database (see section *Study Design and Setting* above).

Participants will be excluded if (1) their child has another chronic pulmonary disease (eg, cystic fibrosis) and (2) parents have insufficient knowledge of French or English to complete the questionnaires.

Potential participants will be identified by a research assistant through prescreening of clinical patient lists. Specifically, the research assistant will prescreen patient lists for the following month and will confirm patient lists using physicians’ daily appointment roster upon arrival at the clinic on each day of recruitment.

### Recruitment

We will recruit 200 families, stratified to recruit 40 families geographically from each quintile of the Pampalon material deprivation index [65-67]. Refer to the *Power Calculation and Feasibility* section for the method of determining the sample size. The Pampalon material deprivation index, measured at the level of the dissemination area and associated with the postal code of the child’s primary residence, is a composite of the (1) proportion of persons without a high school diploma, (2) employment:population ratio, and (3) average personal income obtained from the Canadian census [65,66].

Previous evidence suggests that adherence decreases with the age of the child, coinciding with responsibility for taking medication shifting from parents to their children as they age [53-59], whereas evidence for the impact of children’s sex is mixed [56,57,62,68,69]. We will stratify our recruitment to ensure equal recruitment between age groups (2-7, 8-12, and 13-17 years), and all analyses will consider the child’s age and sex [4].

### Consent

Informed consent will be obtained from all participants in the asthma clinic, before enrollment, including consent for follow-up measures and access to clinical data in the reMed database. Consent forms and questionnaires will be administered in French and English according to each participant’s preference.

Participants will be approached by a trained research assistant at the time of their clinic appointment. Given the patient flow and clinic organization, participants may be approached in the waiting room before their appointment, between parts of their appointment (ie, after seeing the nurse and before seeing their physician), or after having completed their appointment. The research assistant will approach the patients and describe the project and what their participation would require. Those who express interest in participating will accompany the research assistant to a private room where they will be given the opportunity to read the project consent form, with the research assistant available as needed to explain further or respond to questions. Participants who agree to participate will be asked to sign and date the consent form (either on paper or electronically using the REDCap [Research Electronic Data Capture; Vanderbilt University] database where the survey is hosted) as well as complete the consent form and questionnaire for inclusion in the reMed database, which is required for study participation (given that adherence to medication, obtained from this database, is the primary outcome). Participants will then complete the questionnaire on the REDCap database with guidance from the research assistant throughout.

### Follow-up

**Figure 1:**
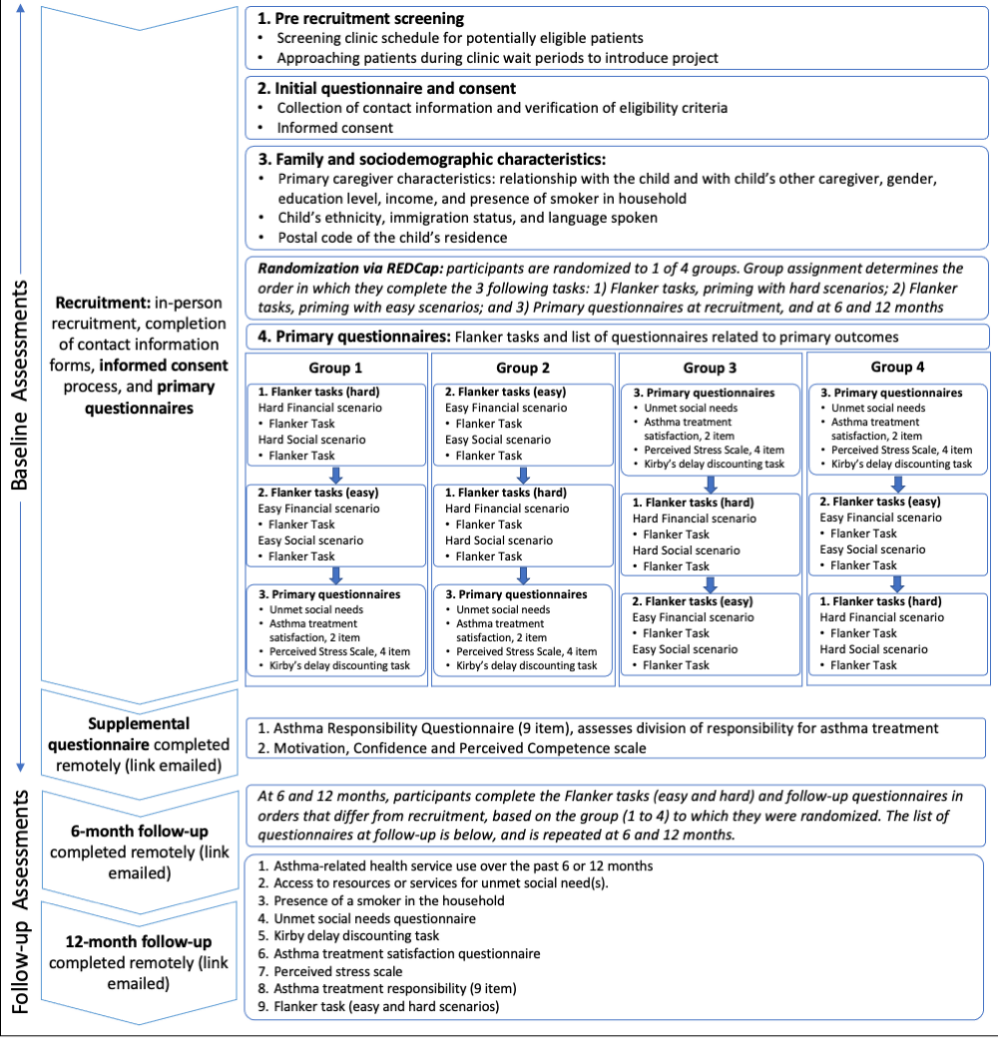
Data collection and schedule of events. REDCap: Research Electronic Data Capture.

Participants will be followed prospectively for 12 months after recruitment with measurements at baseline, 6 months, and 12 months (Figure 1). Using the REDCap database, they will be sent the follow-up questionnaires immediately following the baseline questionnaire and at the 6- and 12-month follow-ups. Refer to Figure 1 for the details of each measure.

### Outcome Measures

Details of each measure, including operational definitions and data collection methods, are described in the following sections (*Primary Outcome*, *Main Predictors*, *Other Measures and Covariates*, and *Follow-up Measures* sections) and detailed in Table 1.

**Table 1:** Overview of measures, data sources, and timeline of measures.

Measure		Measurement details	Data source	Timeline		
				Recruitment	6-month follow-up	12-month follow-up
**Primary outcome**						
	*Adherence*^a^*:* Proportion of prescribed days covered	Number of days for which the drug was dispensed by a pharmacy divided by the number of days for which it was prescribed [63,70]	Pharmacy claims data (*Registre de données sur les Médicaments*)	—^b^	—	X^c^
**Main independent variables**						
	*Unmet social needs*	A series of validated questions assessing employment [71], housing stability and ability to pay for utilities, [72-75], financial resource strain [76-78], food security [79], transportation [75], childcare [80], and parent education and health literacy [80,81]	Questionnaire	X	X	X
	*Scarcity, via executive control—*Eriksen flanker task	Speed of response and percentage accuracy [82-84], Δ accuracy between easy and hard condition	In-person computer-based task	X	X	X
	*Future discounting—*Kirby delay discounting task	27-item questionnaire assessing temporal discounting	Questionnaire	X	X	X
**Exploratory measures**						
	*Capability*	Perceived Competence Scale [85,86]	Questionnaire	X	—	—
	*Motivation*	Treatment Self-Regulation Scale (adapted for adherence) [87]	Questionnaire	X	—	—
	*Perceived stress:* perceived stress scale	4-item measure used to understand how respondents perceive stress and how their feelings are impacted by certain situations [88]	Questionnaire	X	X	X
	*Asthma severity*: PPACI^d^	4-level PPACI assessed in the preceding 6-month period. On the basis of the number of doses per week of short-acting beta-agonists, use of oral corticosteroids within 14 days of a medical claim or hospitalization for asthma, any ED visit for asthma, and any hospitalization for asthma [89]	Medical chart	X	X	X
	*Access to resources*	Whether families accessed resources to which they were referred (social worker at local community health center)	Questionnaire	—	X	X
	*Asthma treatment satisfaction*	Single item inquiring who is primarily responsible for the child’s asthma management and 1 question drawn from the Asthma Medication questionnaire [90] adapted to inquire specifically about management of one’s child’s asthma	Questionnaire	X	X	X
	*Asthma responsibility questionnaire*	10-item measure examining the division of asthma management responsibilities between child and parent [91]	Questionnaire	X	—	X
**Covariates**						
	Child’s *age* and *sex*	N/A^e^	Medical chart	X	—	—
	Child’s *gender*	N/A	Questionnaire	X		
	*Caregiver’s relationship with child*	Two items interrogating relationship to the child and whether respondent is the child’s legal guardian	Questionnaire	X	—	—
	*Caregiver’s gender*	N/A	Questionnaire	X	—	—
	*Caregiver’s marital status* and *living situation*	Single items assessing marital status and living situation as it relates to the child	Questionnaire	X	—	—
	*Postal code of the child’s residence*	N/A	Questionnaire	X	—	—
	*Caregiver’s level of education*	Elementary school or less, some high school, high school graduate, some college or technical school, college graduate, or completed a graduate degree	Questionnaire	X	—	—
	*Household income*	One item question based on the Canadian income survey [92]	Questionnaire	X	—	—
	*Presence of a smoker or e-cigarette user in the house*	2 question items investigating use of tobacco products or e-cigarettes in the last month [93]	Questionnaire	X	X	X
	*Responsibility for asthma management*	“Who is responsible for asthma management?”	Questionnaire	X	—	—
	*Child’s ethnicity, immigration status*, and *language spoken at home*	Questions using categories from Canadian census [94]	Questionnaire	X	—	—
	*Class of controller medication received during study period*	Inhaled corticosteroids and long-acting beta-agonists of leukotriene receptor antagonists	Medical chart	X	X	X

^a^The name of the variable in question has been italicized.

^b^Em dashes in the timeline signify that data for the associated variable will not be collected at the time point in question.

^c^X in the timeline indicate data collection for the associated variable at the time point (recruitment, 6-month follow-up, or 12-month follow-up) in question.

^d^PPACI: Pediatric Pharmacoepidemiology Asthma Control Index.

^e^N/A: not applicable.

### Primary Outcome

*Adherence to controller medication* will be measured for the 12 months of follow-up on a scale of 0%-100% by the *proportion of prescribed days covered (PPDC)*, a validated index calculated as the number of days for which the drug was dispensed by a pharmacy divided by the number of days for which it was prescribed [63]. The PPDC accounts for varying durations of physician prescriptions [70] and leads to conservative estimates of adherence, as possession of medication is a prerequisite for adherence but does not confirm its use. The PPDC will be calculated using medical records (prescriptions made by the clinician) and the reMed database [24,63]. reMed collects drug claims for both privately and publicly insured children.

### Main Predictors

**Figure 2:**
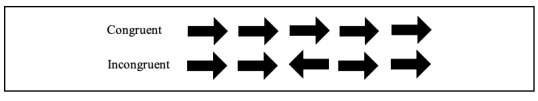
Flanker task with arrows, illustrating congruent and incongruent series of arrows.

*Scarcity* is a state in which individuals’ cognitive resources (mental bandwidth) are occupied and taxed by preoccupations, including pressing financial concerns [31]. These worries consume mental bandwidth, leaving less of it available for other tasks. In this study, we will assess scarcity as per the original work in the field [95]. Briefly, participants will be exposed to hypothetical scenarios that are designed to elicit a scarcity mindset in those who are experiencing a state of scarcity. The scenarios bring to the fore participants’ scarcity mindset by asking participants to imagine themselves in situations in which they would need to access scarce resources. Specifically, participants will be asked to imagine requiring an urgent car repair, costing either US $1500 in the more taxing, “hard” scenario or US $150 in the “easy” scenario. Similarly, participants will be asked to imagine that their child required either a hospitalization (hard scenario) or an ED visit (easy scenario) for their asthma. After listening to the scenarios, participants will complete the Eriksen flanker task, a widely used, standardized, and validated test of executive function assessing selective attention, information processing, and inhibitory function. The task requires participants to rapidly identify whether a series of arrows, colored boxes, or letters are congruent or incongruent (Figure 2). We will measure both the percentage accuracy and time to complete the task under each condition [31,82,95]. The outcome for each participant will be the difference in accuracy on the flanker test after the easy versus the hard scenarios.

*Future discounting* will be assessed using Kirby delay discounting task, in which participants are asked to choose between a smaller, immediate financial reward or a larger, delayed reward. From the participant’s answers to the 27 choices, a hyperbolic discount parameter (k) is calculated [96].

*Unmet social needs* will be assessed using validated measures of employment [71], housing stability, ability to pay for utilities [72-75], financial resource strain [76-78], food security [79], transportation [75], childcare [80], parent education, and health literacy [80,81]. We chose these needs as they had published and validated short question items assessing them, had either documented or plausible relationships with asthma, and could be amenable to intervention by social workers.

Families for whom unmet social needs have been identified will be asked whether they are interested in receiving support to address their needs. If they are interested, the study team will ensure referral to a social worker at a *centre locaux de services communautaires* (local community services centers), a service covered by the government’s universal health insurance plan. With the participants’ consent, their treating clinician will be informed of their unmet social needs and whether they were referred to a local community services center. Participants who agree to be referred to resources to address their unmet social needs at recruitment will be asked at 6 and 12 months if they accessed any resources to help them address their unmet needs and, if so, which ones. In our analysis, among participants with identified unmet social needs, we will explore whether the participants’ desire for a referral for social services and, if applicable, access to those resources influenced the relationship between unmet social needs and adherence.

### Other Measures and Covariates

*Family and sociodemographic characteristics* will include the child’s age, sex, gender, ethnicity, and immigration status and parent participants’ gender, race and ethnicity, level of education, and income. We will also inquire about the presence of a smoker in the household and the languages spoken at home.

*Asthma and asthma treatment information* will include controller medication type (inhaled corticosteroids, long-acting beta-agonists, or leukotriene receptor antagonists) and frequency (once vs twice daily) retrieved from medical records and asthma disease severity in the 6 months preceding recruitment using the 4-level Pediatric Pharmacoepidemiology Asthma Control Index (PPACI) using data from medical records. The PPACI categorizes asthma control based on the average number of doses per week of short-acting beta-agonists, any use of oral corticosteroids within 14 days of a medical claim or ED visit for asthma, and any hospitalization for asthma [89].

*Perceived efficacy of medication* will be assessed using a single item on a 5-point scale selected from the Patient Satisfaction with Asthma Medication questionnaire: “Would you recommend your child’s asthma medication and its inhaler to someone else with asthma?” [90].

*Perceived stress of respondents* will be measured using the 4-item version of the Perceived Stress Scale, with each question rated on a 5-point scale [88,97]. The 4-item Perceived Stress Scale has a Cronbach α reliability coefficient of .72.

*Capability and Motivation* will be measured using a questionnaire based on the Capability, Opportunity, Motivation, and Behavior model [98]. This will serve to evaluate the mechanism by which scarcity and future discounting may influence medication adherence.

*Capability* will be measured using the 4-point Perceived Competence Scale, validated in adherence to medication as well as in the pediatric population [85,86]. These 4 items inquire specifically about the parent’s perceived confidence and capability in ensuring that their child takes their asthma medication as prescribed.

*Motivation* will be assessed using selected items from the 15-item Treatment Self-Regulation Scale adapted for adherence [87]. The 2 selected items assess the perceived importance that their child takes their asthma medication as prescribed and their confidence in ensuring that they do so. Selected items, rather than the full scale, were chosen to limit participant burden.

*Division of asthma treatment responsibility* will be assessed using the 10-item Asthma Responsibility Questionnaire, which measures the division of asthma management responsibilities between child and parent [91]. It also measures the parent’s degree of satisfaction with the amount of responsibility their child takes for their asthma.

### Follow-up Measures

At 6 and 12 months, we will assess health service use, specifically asthma-related ED visits and hospitalizations, via parental reports as well as the patient’s hospital file and provincial health insurance data. We will also measure access to resources to address unmet social needs for participants who agreed to be referred to a social worker at their local community services center using a single-item question for each identified unmet social need (eg, “Since you entered this study, have you been in contact with a social worker or someone from the local community services centre to help you access resources for [identified unmet social needs]?”)

### Study Procedures

Families will be made aware of the project via a posting in the clinic’s regular electronic newsletter and posters in the clinic waiting room. Clinicians will be informed about the study through personalized emails from the study team. A research assistant will be present during each clinic day, prescreen clinic lists for eligible families, and approach primary caregivers for consent at the time of their clinic visit.

### Study Assessments

The study flow, including the measurements at each time point, is shown in Figure 1. Measures will be completed on the web via the REDCap platform [99], except for the flanker task, which will be administered via the testable web-based platform.

### Baseline

Potential participants will be approached by the research assistant during clinic hours, before their child’s appointment with their treating physician. After ensuring that participants are eligible based on the inclusion and exclusion criteria, participants who consent to the study will be randomized to 1 of 4 groups, which determines the order in which they will complete the baseline questionnaire, the flanker task while primed with easy scenarios, and the flanker task while primed with hard scenarios. Details of the questionnaire sequences and randomization groups are shown in Figure 1. The participants will complete the baseline questionnaire on site guided by the research assistant. This includes the following measures (detailed in the *Primary Outcome*, *Main Predictors*, *Other Measures and Covariates*, and *Follow-up Measures* sections above): (1) scarcity assessment (flanker task)—2 rounds, once while primed with “hard” scenarios and once while primed with “easy” scenarios; (2) family and sociodemographic characteristics; (3) unmet social needs; (4) identification of person primarily responsible for the management of the child’s asthma and satisfaction with asthma treatment; (5) Perceived Stress Scale; and (6) Kirby delay discounting task to assess future discounting.

To ascertain whether exposure to the scarcity-inducing scenarios and the subsequent completion of the flanker task impact self-reporting of unmet social needs, participants will be randomized to complete the flanker task either before or after having completed the other sections of the questionnaire. Similarly, to determine whether familiarity with the flanker test influences accuracy and speed rather than the type of scenario (easy vs hard), participants will be randomized to start with either the easy or the hard scenarios. A visual representation of the 4 permutations is presented in Figure 1.

### Secondary Questionnaire

Immediately after the recruitment visit, participants will be emailed a secure link for a secondary questionnaire including (1) the Asthma Responsibility Questionnaire [91] and (2) questions drawn from the Perceived Competence Scale [85,86] and the Treatment Self-Regulation Scale (adapted for asthma treatment adherence) [87]. This division of the baseline and secondary questionnaire was chosen to limit participant burden and time required at the clinic on the day of recruitment.

### Follow-up Questionnaires

Participants will be emailed 6 and 12 months after recruitment to complete the follow-up questionnaires, the contents of which are described in Table 1. Participants will be reminded and offered help to complete the questionnaire if not completed within a week via up to 2 email reminders and 1 phone call from the research assistant.

### Participant Compensation

Participants will receive CAD $10 (US $7.50) in compensation for completion of the initial study questionnaire and CAD $10 (US $7.50) at the end of the study if all materials have been completed.

### Statistical Analysis

Results will be reported following the recommendations of the Strengthening the Reporting of Observational Studies in Epidemiology Statement for cohort studies [100]. For *aim 1*, we will first perform descriptive statistics for our measures of executive function (accuracy and time to complete the Eriksen flanker task between easy and hard scenarios) at baseline, 6 months, and 12 months and model trajectories using mixed effects regression models. We will then quantify the relationship between both level of scarcity at baseline and trajectories with PPDC in the 12 months following the index visit. We will use unadjusted linear regression models, before adjusting for potential confounders, including sociodemographic factors (eg, child age and sex) and disease characteristics (eg, PPACI at baseline and class and dosing frequency of controller medication). We will perform collinearity diagnostics on potential confounders, and if significant correlation is identified, we will adjust our model accordingly. Exploratory analyses will use logistic and ordinal regression models and the same set of variables to examine any effect of scarcity on health service use (ED visit or hospitalization). We will use the same analysis plan to examine the relationship between future discounting (using Kirby’s discount parameter) and PPDC.

For *aim 2*, we will first assess whether scarcity and future discounting scores at baseline differ between families with versus those without unmet social needs. In the subgroup with identified unmet social needs at any time during the study, we will use regression models with time-varying exposure to estimate the effect of referral to social services and access to services on the presence of unmet social needs (ie, determine if referral to social services led to improvement in unmet social needs). We will then use generalized estimating equations to estimate the effect of unmet social needs on cognitive capacity, executive control, and future discounting.

For *aim 3*, we will evaluate whether scarcity is a mediator of the relationship between unmet social needs and adherence with multiple regression models using a recognized approach [101]. We will use a similar analysis for future discounting.

For *aim 4*, we will use multivariable logistic regression models to examine the effect of unmet social needs on ED visits (any vs none) and hospitalizations (any vs none), testing whether there is an interaction between medication adherence and unmet social needs in this relationship (whether better adherence is protective in children with unmet social needs). A similar approach will be used for asthma control using an ordinal regression model for the 4-point PPACI.

For *aim 5*, we will use descriptive statistics to document the prevalence of unmet social needs in our sample as well as within families and use logistic regression models to test whether referrals to social services modulate those trajectories.

### Power Calculation and Feasibility

Previously published data using the asthma clinic database found a mean PPDC (our measure of adherence and primary outcome) of 68%, with an SD of 33% [24]. In previous studies, a 25% [102] or 1 SD difference [103] in adherence was associated with significant differences in the number of ED visits. As those studies used slightly different measures of adherence, we will be conservative and power our study to detect a 16% difference (half an SD) in PPDC between the participants above and below the median of scarcity and future discounting scores. Using 2-sample *t* tests (2-tailed), an α of .05, and a power of 0.8, this will require us to recruit 135 participants. Accounting for 30.4% (41/135) of missing data (covariates) and loss to follow-up, we will recruit 200 participants. The prevalence of unmet social needs in the clinical population is unavailable; however, in our patient population, 26.59% (335/1260) of children are from the lower quintile of the material deprivation index. In addition, recent Canadian data suggest a food insecurity prevalence of 29% among youth and young adults [104]. Assuming that 25% (50/200) of participants have at least one unmet social need, the proposed sample size will allow 85% power to detect a 16% difference (half an SD) in adherence between those with versus without unmet social needs.

On the basis of preliminary data from the asthma clinic, 87.68% (1139/1299) of patients who were prescribed daily controller medications consented to reMed. In another study (Poitras N et al, unpublished data, August 2023) from our clinic with a similar population and participant burden, 30% of eligible families consented to participate in the study. Using this ratio, we would need to approach approximately 670 families to obtain our desired sample size. There are at least three half-day clinics a week where recruitment could occur, with a minimum of 3 families per clinic. Using a conservative estimate, even if 29.9% (200/670) of families consent to participate, recruiting 200 participants will take 18 months.

## Results

This study obtained funding in April 2021. The research activities of this study began in December 2021. Participant enrollment and data collection began in August 2022 and are expected to be completed in 24 months (August 2024). As of February 2023, 100 participants have been recruited and have completed all baseline data. Data analysis will begin once all participants have been recruited and follow-up measures (6-month and 12-month follow-up) are in progress. Primary results of the study are expected to be available in the first months of 2025.

## Discussion

### Principal Findings

This project will allow us to document the impact of unmet social needs, scarcity, and future discounting on adherence in a diverse population of children with asthma using robust documentation of adherence and validated measures of scarcity and future discounting. The study will also allow us to document ongoing changes in both unmet social needs and adherence to medication in children with asthma in the wake of the COVID-19 pandemic. This research will confirm the potential for scarcity and future discounting as novel targets for either new interventions or improving existing effective interventions to improve adherence to controller medication that may lead to reduced risk across the life course for vulnerable children with asthma. The development of an intervention and assessment of its effectiveness through a randomized trial will be the next stages of this line of research, with potential benefits at both the national and international levels (including in low- and middle-income countries) to ultimately help reduce the disease burden in this population.

### Strengths

TTo the best of our knowledge, this study is the first to investigate the relationship between unmet social needs, scarcity, and future discounting in medication adherence. We will use objective measures of adherence, validated questionnaires, and psychometric testing to measure the main independent variables of interest.

We will recruit patients from a clinic with diverse population, representing a broad range of demographic characteristics, including SES and social situations, which we will be able to capture in detail with both our unmet social needs questionnaires and demographic questions. This will allow for important subgroup analyses by age, sex, race and ethnicity, and SES.

Finally, we are performing this study in Canada, where medical care insurance is universal and government-funded and where prescription medication insurance is also universal (either privately or publicly funded). As such, it will help better understand the factors influencing adherence in a setting where financial barriers to either care or prescription medication are lower, especially for low-income populations.

### Limitations

#### Representativeness of the Population

Recruitment from a tertiary pediatric center clinic could, in theory, limit generalizability; however, the clinical population is quite diverse with regard to SES, race, and ethnicity [105]. Previous studies reported lower rates of controller medication prescription in patients with lower SES [106], leading to a potential selection bias. We will limit this bias through stratified recruitment by SES and an analysis of all eligible patients approached to ensure that a similar proportion of patients in each SES stratum were excluded owing to a lack of controller medication prescription.

#### Potential Biases

By collecting objective values of adherence using reMed, we will avoid self-reporting bias in adherence, which is our primary outcome. It is possible that objective adherence will be positively influenced by the mere involvement in the research study (Hawthorne effect). Would that occur, it would likely be of similar magnitude for all participants in the study and not substantially influence the associations found. We will also be able to estimate the size of this effect by comparing adherence in this study with previously reported historical data on adherence from other children followed in the clinic [105].

Social desirability may influence parents’ report of confidence in managing their child’s treatment or other elements on the questionnaire. We attempted to mitigate this potential bias through the selection of previously validated questionnaires as well as an emphasis on the nonjudgmental nature of the questionnaire and the confidentiality of the information they provide. Again, this potential bias would likely be present across our sample and therefore should not influence the relationship found in the study.

### Conclusions

Documenting the impact of unmet social needs, scarcity, and future discounting on adherence in a diverse population of children with asthma will provide important data to allow for improved approaches to prescribing and monitoring asthma treatment adherence in children. Understanding and targeting elements especially prevalent in this vulnerable population also has the potential to lead to significant health care cost savings in addition to reducing health inequalities by improving outcomes for populations at highest risk. Finally, our methodological approach to examine scarcity and future discounting and their link to medication adherence has the potential to be applied to a wide range of other chronic diseases (diabetes, cystic fibrosis, heart disease, etc), potentially leading to improvements in health and quality of life for children and adults living with chronic diseases.

## Appendix

Multimedia Appendix 1 Peer review report from the Psychosocial, sociocultural and behavioral determinants of health 2 / Déterminants psychosociaux, socioculturels et comportementaux de la santé 2 - Canadian Institutes of Health Research/Instituts de recherche en santé du Canada.

## Abbreviations

ED: emergency department
PPACI: Pediatric Pharmacoepidemiology Asthma Control Index
PPDC: proportion of prescribed days covered
REDCap: Research Electronic Data Capture
reMed: Registre de données sur les Médicaments (data registry for prescribed medications)
SES: socioeconomic status

## Data Availability

Data for this project will be made available following the conclusion of the study from the corresponding author upon reasonable request for use for noncommercial purposes.
