# Supplementary material for: Impact of Unmet Social Needs, Scarcity, and Future Discounting on Adherence to Treatment in Children With Asthma: Protocol for a Prospective Cohort Study
Source: JMIR Res Protoc. 2023 Mar 7;12:e37318. doi: 10.2196/37318 (PMC10131837; doi:10.2196/37318)
Supplement: Multimedia Appendix 1 [file resprot_v12i1e37318_app1.pdf]

July/Juillet 15, 2021
DCO150GP

Canadian Institutes of Health Research/Instituts de recherche en santé du Canada
Notice of Recommendation/Avis de recommandation

Application Number/Numéro de la demande: 461392
Committee Code/Code du comité: PB2

Applicants/Candidats: Docteur Olivier Drouin

With/Avec: Dr. T. Barnett, Prof. F. Ducharme, Dr. E. Fleegler, Dr. K. Lavoie, Docteur S. Sultan, Dr. S. Tse

Institution paid/ Établissement payé: Centre hospitalier universitaire Sainte-Justine (Montréal, Québec)

Title/Titre: Improving adherence to controller medication in children with asthma - the unexplored contribution of behavioural sciences

Primary Inst./Inst. principal: Développement et santé des enfants et des adolescents
Other Related Inst./ Autres inst. connexes: Santé circulatoire et respiratoire; Santé publique et des populations

Competition /Concours: Subvention Projet
April/Avril 01, 2021

Number in competition/Nbre de demandes dans le concours: 2381

Peer Review Committee Recommendation, for your information and use/
Recommandation du comité d'examen par les pairs, pour fins d'information et d'utilisation:

**Committee/Comité:**	Déterminants psychosociaux, socioculturels et comportementaux de la santé 2
**Number reviewed/ Demandes examinées:**	23
**Application rank within the committee/ Rang de la demande dans le comité:**	1
**Percent Rank within the committee / Rang en pourcentage au sein du comité:**	100%
**Rated / Cote:**	4.56
**Recommended Term/ Durée recommandée:**	4 years/ans 0 months/mois
**Recommended average annual operating amount/ Montant annuel moyen recommandé pour le fonctionnement:**	$123,750
**Recommended equipment amount/ Montant recommandé pour les appareils:**	$0

This document is for information only.
An application rated below 3.50 is ineligible for CIHR funding. For applications rated 3.50 and above, please note that it is the application's rank within the peer review committee that determines whether it is funded, rather than its absolute rating. The final funding decision will be communicated in the Notice of Decision.

Document à titre d'information seulement.
Une demande cotée en dessous de 3,5 n'est pas admissible au financement des IRSC. En ce qui a trait aux demandes cotées 3,50 ou plus, veuillez noter que l'on détermine l'attribution des fonds en fonction du classement obtenu au sein du comité d'examen par les pairs plutôt qu'en fonction du classement absolu. La décision finale relative au financement sera communiquée dans l'Avis de décision.

July/Juillet 22, 2021

DC0190GP

Canadian Institutes of Health Research / Instituts de recherche en santé du Canada

Notice of Decision / Avis de décision

Application Number/Numéro de la demande: 461392
Committee Code/Code du comité: PB2

Applicants/Candidats:	Docteur Olivier Drouin			
With/Avec:	Dr. T. Barnett	Prof. F. Ducharme	Dr. E. Fleegler	Dr. K. Lavoie
	Docteur S. Sultan	Dr. S. Tse		
Institution paid/	Centre hospitalier universitaire Sainte-Justine (Montréal, Québec)			
Title/Titre:	Improving adherence to controller medication in children with asthma - the unexplored contribution of behavioural sciences			
Primary Inst./ Inst. principal:	Human Development, Child and Youth Health / Développement et santé des enfants et des adolescents			
Other Related Inst./ Autres inst. connexes:	Circulatory and Respiratory Health / Santé circulatoire et respiratoire; Population and Public Health / Santé publique et des populations			

**Competition Outcome/Résultats du concours:**	Project Grant / Subvention Projet<br>April/Avril 01, 2021
**Number in competition/Nbre de demandes dans le concours:**	2381
**Number approved/Nbre de demandes approuvées:**	422

**Decision on your application/ Décision sur votre demande:**	Approved / Approuvée
**Total Funding Amount:/ Montant total du financement:**	$378,675
**Term/Durée:**	4 **yrs/ans** 0 **months/mois**

**Peer Review Committee Recommendation, for your information and use/ Recommandation du comité d'examen par les pairs, pour fins d'information et d'utilisation:**	
**Committee/Comité:**	Psychosocial, Sociocultural & Behavioural Determinants of Health 2 / Déterminants psychosociaux, socioculturels et comportementaux de la santé 2
**Number reviewed/ Nbre de demandes examinées:**	23
**Number approved in that committee/ Nbre de demandes approuvées dans ce comité:**	4
**Application rank within the committee/**	1
**Percent Rank Within the Committee/**	100%
**Rating/**	4.56

**Additional Funding Opportunities/ Opportunités de financement add**	**Decision/ Décision**	**Total Funding Amount/Montant total du financement**	**Competition Code/Cote de conc**	**Application Number/ Numéro de la demande**
Project Grant - PA: Human Development, Child and Youth Health/Subvention Projet - AP: Développement et santé des enfants et des adolescents	Non approuvée/ Not Approved	$0	202104PJA	465339
Project Grant - PA: Patient-Oriented Research: Early-Career Investigator/Projet - AP : Recherche axée sur le patient : Chercheur en début de carrière	Non approuvée/ Not Approved	$0	202104PJM	465340
Project Grant - Priority Announcement: Population and Public Health/Subvention Projet - Annonce de priorité : Santé publique et des populations	Non approuvée/ Not Approved	$0	202104PAA	465341

This document is for information only. Official payment is stated on the CIHR Authorization for Funding.
Document à titre d'information seulement. Le paiement officiel est indiqué sur l'autorisation de financement des IRSC.

Canadian Institutes of Health Research
160 Elgin Street, 9th Floor
Address Locator 4809A
Ottawa, Ontario K1A 0W9

Instituts de recherche en santé du Canada
160, rue Elgin, 9e étage
Indice de l'adresse 4809A
Ottawa (Ontario) K1A 0W9

Institute of Aging
Institute of Cancer Research
Institute of Circulatory and Respiratory Health
Institute of Gender and Health
Institute of Genetics
Institute of Health Services and Policy Research
Institute of Human Development and Child and Youth Health
Institute of Indigenous Peoples' Health
Institute of Infection and Immunity
Institute of Musculoskeletal Health and Arthritis
Institute of Neurosciences, Mental Health and Addiction
Institute of Nutrition, Metabolism and Diabetes
Institute of Population and Public Health

Institut du vieillissement
Institut du cancer
Institut de la santé circulatoire et respiratoire
Institut de la santé des femmes et des hommes
Institut de génétique
Institut des services et des politiques de la santé
Institut du développement et de la santé des enfants et des adolescents
Institut de la santé des Autochtones
Institut des maladies infectieuses et immunitaires
Institut de l'appareil locomoteur et de l'arthrite
Institut des neurosciences, de la santé mentale et des toxicomanies
Institut de la nutrition, du métabolisme et du diabète
Institut de la santé publique et des populations

Le 22 juillet 2021

Docteur Olivier Drouin
CHU Sainte-Justine
Service de pédiatrie générale
3175 Chemin de la Côte-Sainte-Catherine
7e étage, Bloc 9
Montreal, Québec H3T 1C5

Docteur Drouin,

Au nom des Instituts de recherche en santé du Canada (IRSC), j'ai le plaisir de vous informer que votre demande intitulée « Improving adherence to controller medication in children with asthma - the unexplored contribution of behavioural sciences », présentée au concours de subventions Projet du printemps 2021, a été approuvée pour du financement.

Les évaluations et les résultats de votre demande sont accessibles dans RechercheNet. Si vous ne pouvez accéder aux documents, veuillez communiquer avec nous à support-soutien@cihr-irsc.gc.ca. Votre autorisation de financement vous sera envoyée par courriel.

Il convient de souligner que le nombre de demandes approuvées (indiqué dans l'avis de décision) comprend les demandes figurant au-dessus du seuil de financement établi pour le comité et, le cas échéant, les demandes financées dans le cadre du processus de rééquilibrage tel que décrit dans Le processus décisionnel du Programme de subventions Projet sur notre site Web (https://cihr-irsc.gc.ca/f/52317.html). Le processus de rééquilibrage est en place afin que la proportion de subventions financées au sein des cohortes admissibles au rééquilibrage soit au moins égale à celle des demandes soumises par les cohortes admissibles au concours.

Étant donné que les IRSC n'informent pas les cocandidats de leur décision, nous vous prions de communiquer le résultat de cette demande aux personnes concernées et à leurs établissements de recherche (s'ils diffèrent du vôtre).

Pour toute question, n'hésitez pas à communiquer avec un agent de traitement du centre de contact par téléphone au 613-954-1968 ou par courriel à support-soutien@cihr-irsc.gc.ca.

Nous vous félicitons pour votre réussite à ce concours.

Sincères salutations,

C. Leneï

Chaidwick Leneïs
Gestionnaire, Conception et exécution des programmes
Portefeuille des programmes de recherche

500517-202104PJT-PB2-461392-136118-DLPJT

CIHR IRSC
Discoveries for life / Découvertes pour la vie

Canada

Canadian Institutes of Health Research
160 Elgin Street, 9th Floor
Address Locator 4809A
Ottawa, Ontario K1A 0W9

Instituts de recherche en santé du Canada
160, rue Elgin, 9e étage
Indice de l'adresse 4809A
Ottawa (Ontario) K1A 0W9

Institute of Aging
Institute of Cancer Research
Institute of Circulatory and Respiratory Health
Institute of Gender and Health
Institute of Genetics
Institute of Health Services and Policy Research
Institute of Human Development and Child and Youth Health
Institute of Indigenous Peoples' Health
Institute of Infection and Immunity
Institute of Musculoskeletal Health and Arthritis
Institute of Neurosciences, Mental Health and Addiction
Institute of Nutrition, Metabolism and Diabetes
Institute of Population and Public Health

Institut du vieillissement
Institut du cancer
Institut de la santé circulatoire et respiratoire
Institut de la santé des femmes et des hommes
Institut de génétique
Institut des services et des politiques de la santé
Institut du développement et de la santé des enfants et des adolescents
Institut de la santé des Autochtones
Institut des maladies infectieuses et immunitaires
Institut de l'appareil locomoteur et de l'arthrite
Institut des neurosciences, de la santé mentale et des toxicomanies
Institut de la nutrition, du métabolisme et du diabète
Institut de la santé publique et des populations

Le 22 juillet 2021

Docteur Olivier Drouin
CHU Sainte-Justine
Service de pédiatrie générale
3175 Chemin de la Côte-Sainte-Catherine
7e étage, Bloc 9
Montreal, Québec H3T 1C5

Bonjour,

Au nom des Instituts de recherche en santé du Canada (IRSC), j'aimerais vous féliciter de votre succès au récent concours de financement des IRSC!

En tant que chercheurs du domaine de la santé, nous partageons un objectif commun : améliorer la santé et le bien-être des populations, au Canada et dans le monde. Par l'entremise des IRSC, le gouvernement du Canada offre un soutien essentiel aux chercheurs canadiens œuvrant dans tous les thèmes de la recherche en santé, qui sont étroitement liés, afin d'améliorer la santé de toute la population canadienne. Titulaire d'une subvention des IRSC, vous faites maintenant partie de cette entreprise.

Vous n'êtes pas sans savoir que l'évaluation de votre demande de subvention a été possible grâce aux pairs évaluateurs qui ont généreusement donné de leur temps pour soutenir le secteur canadien de la recherche en santé. À titre de titulaire de fonds des IRSC, vos connaissances et votre expertise sont fort précieuses. C'est pourquoi je vous invite à envisager de devenir membre du Collège des évaluateurs, si vous ne l'êtes pas déjà. Veuillez consulter la page www.cihr-irsc.gc.ca/f/49923.html pour prendre connaissance des critères de sélection et pour savoir comment soumettre votre candidature.

Aujourd'hui, l'activité scientifique est surveillée de près et il est plus important que jamais de veiller à ce que notre travail soit bien compris. Je vous demande donc de faire mention du financement des IRSC dans vos présentations et vos communications au sujet de vos recherches, et de continuer de faire valoir l'importance cruciale de la recherche pour la santé des Canadiennes et des Canadiens.

Vous trouverez à l'adresse https://cihr-irsc.gc.ca/f/30789.html des moyens de reconnaître le soutien reçu et découvrirez comment l'équipe des Communications des IRSC peut vous aider à faire connaître votre recherche.

Encore une fois, je tiens à vous féliciter de cet exploit et à vous offrir mes meilleurs vœux de succès dans toutes vos entreprises. Je suivrai avec intérêt l'avancement de vos recherches.

Je vous prie de recevoir mes plus cordiales salutations.

Michael J. Strong, M.D., FRCPC, FAAN, MACSS
Président

500518-202104PJT-PB2-461392-136118-CONGR

CIHR IRSC
Discoveries for life / Découvertes pour la vie

Canada

**Review Type / Type d'évaluation:**	Reviewer 1 / Évaluateur 1
**Name of Applicant / Nom du chercheur:**	Drouin, Olivier
**Application No. / Numéro de demande:**	461392
**Agency / Agence:**	CIHR/IRSC
**Competition / Concours:**	Project Grant/Subvention Projet
**Committee / Comité:**	Psychosocial, Sociocultural & Behavioural Determinants of Health 2/Déterminants psychosociaux, socioculturels et comportementaux de la santé 2
**Title / Titre:**	Improving adherence to controller medication in children with asthma - the unexplored contribution of behavioural sciences

Adjudication Criteria/Critères de sélection

Initial Score/Cote Initiale: 4.6

Top/Bottom Selection/Groupe supérieur/inférieur

- (●) **Top/Groupe supérieur**
- ( ) **Bottom/Groupe inférieur**

**Review Type / Type d'évaluation:**	Reviewer 1 / Évaluateur 1
**Name of Applicant / Nom du chercheur:**	Drouin, Olivier
**Application No. / Numéro de demande:**	461392
**Agency / Agence:**	CIHR/IRSC
**Competition / Concours:**	Project Grant/Subvention Projet
**Committee / Comité:**	Psychosocial, Sociocultural & Behavioural Determinants of Health 2/Déterminants psychosociaux, socioculturels et comportementaux de la santé 2
**Title / Titre:**	Improving adherence to controller medication in children with asthma - the unexplored contribution of behavioural sciences

Summary of Application/Résumé de la demande:

This study examines medical adherence to asthma controller medications for the treatment of asthma. Study will explore the cognitive factors influencing adherence to medication in children with asthma with a focus on scarcity and future discounting/delayed gratification and unmet social needs and a focus on vulnerable pops

**Review Type / Type d'évaluation:**	Reviewer 1 / Évaluateur 1
**Name of Applicant / Nom du chercheur:**	Drouin, Olivier
**Application No. / Numéro de demande:**	461392
**Agency / Agence:**	CIHR/IRSC
**Competition / Concours:**	Project Grant/Subvention Projet
**Committee / Comité:**	Psychosocial, Sociocultural & Behavioural Determinants of Health 2/Déterminants psychosociaux, socioculturels et comportementaux de la santé 2
**Title / Titre:**	Improving adherence to controller medication in children with asthma - the unexplored contribution of behavioural sciences

Strengths and Weaknesses/Forces et faiblesses:

Strong PI, Medical decision making, pediatrician, asthma specialist, health services research pediatrician and behavioral scientist with demonstrated research capacity, 10 years post-medical school and despite holding an MS (2017) got a MPH from Harvard. Interdisciplinary team of experts from across the country + US collaborators

Study is based on a well articulated theoretical model of trying to understand patient decision making and medical compliance The COM-B (Capability,Opportunity, Motivation, Behaviour)

Very carefully described and designed study to increase adherance for asthma medication

Maximizing the use of secondary data to complement survey data being collected at the clinic

Discuss direct impacts of COVID on treatment adherence but this research also has implications for compliance for other behaviors (eg. COVID vaccinations)

Solid KT plan, academic publications and conferences, electronic newsletter from clinic; website, social media to publicize results

Family partners- CIRCUIT program and will disseminate to families not participating in study, investigators are part of RSRQ Réseau de recherche en Santé Respiratoiredu Québec(RSRQ), a network of 460 respiratory health experts in Québec, teach medical students

Weaknesses

Implication for generalizability of not including new immigrants who don’t speak the language and maybe at increased risk for asthma, access to services, and following physician instructions.

Many children have varying living arrangement (shared custody, multiple living arrangements), consider these as well as adherence by mothers vs fathers for future

Recommendations

Expand KT beyond QC. Several practical messages with possible implications for other chronic diseases(diabetes, cystic fibrosis, heart disease, etc.) and the potential to reduce health care costs, decrease burden on families, and improve quality of life.

**Review Type / Type d'évaluation:**	Reviewer 1 / Évaluateur 1
**Name of Applicant / Nom du chercheur:**	Drouin, Olivier
**Application No. / Numéro de demande:**	461392
**Agency / Agence:**	CIHR/IRSC
**Competition / Concours:**	Project Grant/Subvention Projet
**Committee / Comité:**	Psychosocial, Sociocultural & Behavioural Determinants of Health 2/Déterminants psychosociaux, socioculturels et comportementaux de la santé 2
**Title / Titre:**	Improving adherence to controller medication in children with asthma - the unexplored contribution of behavioural sciences

**Review Type / Type d'évaluation:**	Reviewer 1 / Évaluateur 1
**Name of Applicant / Nom du chercheur:**	Drouin, Olivier
**Application No. / Numéro de demande:**	461392
**Agency / Agence:**	CIHR/IRSC
**Competition / Concours:**	Project Grant/Subvention Projet
**Committee / Comité:**	Psychosocial, Sociocultural & Behavioural Determinants of Health 2/Déterminants psychosociaux, socioculturels et comportementaux de la santé 2
**Title / Titre:**	Improving adherence to controller medication in children with asthma - the unexplored contribution of behavioural sciences

Budget Recommendation/Recommandation budgétaire:

**Review Type / Type d'évaluation:**	Reviewer 1 / Évaluateur 1
**Name of Applicant / Nom du chercheur:**	Drouin, Olivier
**Application No. / Numéro de demande:**	461392
**Agency / Agence:**	CIHR/IRSC
**Competition / Concours:**	Project Grant/Subvention Projet
**Committee / Comité:**	Psychosocial, Sociocultural & Behavioural Determinants of Health 2/Déterminants psychosociaux, socioculturels et comportementaux de la santé 2
**Title / Titre:**	Improving adherence to controller medication in children with asthma - the unexplored contribution of behavioural sciences

Please indicate your appraisal of the integration of sex as a biological variable as a strength, weakness, or not applicable to the proposal./Prière de sélectionner une option pour donner votre évaluation de l'intégration du sexe comme variable biologique en tant que point fort ou point faible de la proposition, ou en tant qu'élément non applicable à la proposition.

- (●) **Strength/Point fort**
- ( ) **Weakness/Point faible**
- ( ) **Not applicable/Non applicable**

Please indicate your appraisal of the integration of gender as a socio-cultural determinant of health as a strength, weakness, or not applicable to the proposal./Prière de sélectionner une option pour donner votre évaluation de l'intégration du genre comme déterminant socioculturel de la santé en tant que point fort ou point faible de la proposition, ou en tant qu'élément non applicable à la proposition.

- (●) **Strength/Point fort**
- ( ) **Weakness/Point faible**
- ( ) **Not applicable/Non applicable**

**Review Type / Type d'évaluation:**	Reviewer 1 / Évaluateur 1
**Name of Applicant / Nom du chercheur:**	Drouin, Olivier
**Application No. / Numéro de demande:**	461392
**Agency / Agence:**	CIHR/IRSC
**Competition / Concours:**	Project Grant/Subvention Projet
**Committee / Comité:**	Psychosocial, Sociocultural & Behavioural Determinants of Health 2/Déterminants psychosociaux, socioculturels et comportementaux de la santé 2
**Title / Titre:**	Improving adherence to controller medication in children with asthma - the unexplored contribution of behavioural sciences

Sex and/or Gender Considerations/Notions de sexe et/ou de genre:

Child and parent sex are considered as important for medication adherence. Parent will complete gender sex role inventory.

**Review Type / Type d'évaluation:**	Reviewer 2 / Évaluateur 2
**Name of Applicant / Nom du chercheur:**	Drouin, Olivier
**Application No. / Numéro de demande:**	461392
**Agency / Agence:**	CIHR/IRSC
**Competition / Concours:**	Project Grant/Subvention Projet
**Committee / Comité:**	Psychosocial, Sociocultural & Behavioural Determinants of Health 2/Déterminants psychosociaux, socioculturels et comportementaux de la santé 2
**Title / Titre:**	Improving adherence to controller medication in children with asthma - the unexplored contribution of behavioural sciences

Adjudication Criteria/Critères de sélection

Initial Score/Cote Initiale: 4.5

Top/Bottom Selection/Groupe supérieur/inférieur

- (●) **Top/Groupe supérieur**
- ( ) **Bottom/Groupe inférieur**

**Review Type / Type d'évaluation:**	Reviewer 2 / Évaluateur 2
**Name of Applicant / Nom du chercheur:**	Drouin, Olivier
**Application No. / Numéro de demande:**	461392
**Agency / Agence:**	CIHR/IRSC
**Competition / Concours:**	Project Grant/Subvention Projet
**Committee / Comité:**	Psychosocial, Sociocultural & Behavioural Determinants of Health 2/Déterminants psychosociaux, socioculturels et comportementaux de la santé 2
**Title / Titre:**	Improving adherence to controller medication in children with asthma - the unexplored contribution of behavioural sciences

Summary of Application/Résumé de la demande:

Asthma affects 10% of children and can lead to serious health consequences. While it can be controlled with medication, adherence is low and this is even more likely to be the case amongst those children with the most social challenges. There is a strong need to increase adherence but research is needed to identify susceptible pathways for change. The applicants suggest that two cognitive mechanisms, scarcity and future discounting, may be important to understanding nonadherence and to mediating the relationship between unmet social needs and medication adherence.

This 12-month prospective observational cohort study will enroll 200 parents whose children have asthma (as well as any of those children who are 8 or older). Recruitment will be stratified by economic situation, sex and age of child and these and parent gender will be considered in all analyses. The study objectives are to understand the relationships between these two cognitive mechanisms and medication adherence, the relationship between having unmet social needs and these cognitive mechanisms, and to test whether scarcity and future discounting are mediators. Parents will consent to have their data from electronic health databases accessed (measure of adherence to controller medication calculated from BAP and reMED using standard procedure, asthma related health visits and hospitalization), complete a baseline, six- and twelve-month assessments. Unmet needs, scarcity and future discounting will be measured at each time point. Referrals will be made at every time point based on reported unmet needs and use/access of new resources will be assessed at subsequent time points. As the COM-B theoretical model suggests they are important to cognitive outcomes, capability and motivation will also be measured. Who is responsible for asthma medication (parent or child or both) will also be measured over time. Children over 8 who agree will complete the Consideration of Future Consequences scale at all three time periods to assess the relationship between parent and child future discounting.

Beyond academic publishing and communication of study results to health care professionals (e.g., respirologists), the KT strategy includes electronic newsletters to families affected by asthma, annual events with families and health care providers, press releases of the findings, and communication with all families who are part of the study through a website and social media.

**Review Type / Type d'évaluation:**	Reviewer 2 / Évaluateur 2
**Name of Applicant / Nom du chercheur:**	Drouin, Olivier
**Application No. / Numéro de demande:**	461392
**Agency / Agence:**	CIHR/IRSC
**Competition / Concours:**	Project Grant/Subvention Projet
**Committee / Comité:**	Psychosocial, Sociocultural & Behavioural Determinants of Health 2/Déterminants psychosociaux, socioculturels et comportementaux de la santé 2
**Title / Titre:**	Improving adherence to controller medication in children with asthma - the unexplored contribution of behavioural sciences

Strengths and Weaknesses/Forces et faiblesses:

STRENGTHS

This is a well constructed and written grant that provides an excellent case for the significance and importance of the topic and need to understand cognitive influences on parent/child adherence to asthma medication. Theoretical and empirical grounding is substantial.

The case is clearly made that social inequality affects medication adherence and must be taken seriously in any attempt to influence change in behaviour. The way that unmet social needs are understood, addressed, and analysed in the proposed study is exemplary. Further, the way this is handled in the study proper, increases confidence that the KT strategy for disseminating findings related to unmet social needs will be supportive and will reduce rather than increase associated stigma (related to poverty, housing or food insecurity, etc.). Response to reviewers, application, and KT plan show involvement of parent partners throughout.

The study design, randomization in within-subjects' factors, measure choices, and procedures are solid and are aligned to meet the study goals. Power analysis shows the sample size is sufficient for testing primary and secondary outcomes. Mitigation strategies are well thought out and timelines are reasonable and achievable.

The NPA is an early career scholar (PhD 2017) with a good grant and publication record. He has demonstrated expertise in this field particularly in studying the influence of social inequities on health behaviours affecting children. He is leading an excellent team of early, mid, and senior career scholars with well defined roles and commitments to the project and deep experience related to marginality and social and health inequalities. The team also involves several individuals who have been instrumental in development of clinical care guidelines which will be invaluable for future KT.

WEAKNESSES

Inclusion of children 8 and over for a single measurement at each time period is interesting but appears outside of the formal Objectives and Aims of the study. If this correlation between parents' and children's future discounting is an important enough contribution to the literature to warrant consenting children, it deserves more formal delineation.

I am concerned that the incentives proposed are so low (just covering parking/transportation) that they may not contribute to reducing attrition. If all measures are being completed on a tablet at all timepoints and require a visit to the clinic for the parent (and in some cases, their child) an incentive of $20 or $25 would be more likely to cover related expenses and to thank them for their time without being coercive. It is not clear where the 30% attrition figure came from, but it is definitely high and may be possible to reduce. This would be important since attrition could be differential particularly related to unmet needs. Higher incentives could help reduce this bias and meet social justice goals of valuing parents' contributions.

**Review Type / Type d'évaluation:**	Reviewer 2 / Évaluateur 2
**Name of Applicant / Nom du chercheur:**	Drouin, Olivier
**Application No. / Numéro de demande:**	461392
**Agency / Agence:**	CIHR/IRSC
**Competition / Concours:**	Project Grant/Subvention Projet
**Committee / Comité:**	Psychosocial, Sociocultural & Behavioural Determinants of Health 2/Déterminants psychosociaux, socioculturels et comportementaux de la santé 2
**Title / Titre:**	Improving adherence to controller medication in children with asthma - the unexplored contribution of behavioural sciences

Budget Recommendation/Recommandation budgétaire:

Budget is appropriate. Concern related to incentives being too low (as explained above).

Please indicate your appraisal of the integration of sex as a biological variable as a strength, weakness, or not applicable to the proposal./Prière de sélectionner une option pour donner votre évaluation de l'intégration du sexe comme variable biologique en tant que point fort ou point faible de la proposition, ou en tant qu'élément non applicable à la proposition.

- (●) **Strength/Point fort**
- ( ) **Weakness/Point faible**
- ( ) **Not applicable/Non applicable**

Please indicate your appraisal of the integration of gender as a socio-cultural determinant of health as a strength, weakness, or not applicable to the proposal./Prière de sélectionner une option pour donner votre évaluation de l'intégration du genre comme déterminant socioculturel de la santé en tant que point fort ou point faible de la proposition, ou en tant qu'élément non applicable à la proposition.

- (●) **Strength/Point fort**
- ( ) **Weakness/Point faible**
- ( ) **Not applicable/Non applicable**

**Review Type / Type d'évaluation:**	Reviewer 2 / Évaluateur 2
**Name of Applicant / Nom du chercheur:**	Drouin, Olivier
**Application No. / Numéro de demande:**	461392
**Agency / Agence:**	CIHR/IRSC
**Competition / Concours:**	Project Grant/Subvention Projet
**Committee / Comité:**	Psychosocial, Sociocultural & Behavioural Determinants of Health 2/Déterminants psychosociaux, socioculturels et comportementaux de la santé 2
**Title / Titre:**	Improving adherence to controller medication in children with asthma - the unexplored contribution of behavioural sciences

Sex and/or Gender Considerations/Notions de sexe et/ou de genre:

Discussion of sex and gender was comprehensive for the topic. Stratification of recruitment by sex of child and inclusion of gender of (the likely mostly women) parents is appropriate.

**Review Type / Type d'évaluation:**	Reviewer 3 / Évaluateur 3
**Name of Applicant / Nom du chercheur:**	Drouin, Olivier
**Application No. / Numéro de demande:**	461392
**Agency / Agence:**	CIHR/IRSC
**Competition / Concours:**	Project Grant/Subvention Projet
**Committee / Comité:**	Psychosocial, Sociocultural & Behavioural Determinants of Health 2/Déterminants psychosociaux, socioculturels et comportementaux de la santé 2
**Title / Titre:**	Improving adherence to controller medication in children with asthma - the unexplored contribution of behavioural sciences

Adjudication Criteria/Critères de sélection

Initial Score/Cote Initiale: 4.5

Top/Bottom Selection/Groupe supérieur/inférieur

- (●) **Top/Groupe supérieur**
- ( ) **Bottom/Groupe inférieur**

**Review Type / Type d'évaluation:**	Reviewer 3 / Évaluateur 3
**Name of Applicant / Nom du chercheur:**	Drouin, Olivier
**Application No. / Numéro de demande:**	461392
**Agency / Agence:**	CIHR/IRSC
**Competition / Concours:**	Project Grant/Subvention Projet
**Committee / Comité:**	Psychosocial, Sociocultural & Behavioural Determinants of Health 2/Déterminants psychosociaux, socioculturels et comportementaux de la santé 2
**Title / Titre:**	Improving adherence to controller medication in children with asthma - the unexplored contribution of behavioural sciences

Summary of Application/Résumé de la demande:

The overall goal of this proposal is to understand the cognitive factors (scarcity, future discounting) influencing controller medication adherence in treating children’s asthma, including an analysis of unmet social need and whether this is mediated by cognitive factors. To accomplish this, the researchers will conduct a 12-month prospective cohort study of 200 families of children aged 2-17 treated daily for asthma.

**Review Type / Type d'évaluation:**	Reviewer 3 / Évaluateur 3
**Name of Applicant / Nom du chercheur:**	Drouin, Olivier
**Application No. / Numéro de demande:**	461392
**Agency / Agence:**	CIHR/IRSC
**Competition / Concours:**	Project Grant/Subvention Projet
**Committee / Comité:**	Psychosocial, Sociocultural & Behavioural Determinants of Health 2/Déterminants psychosociaux, socioculturels et comportementaux de la santé 2
**Title / Titre:**	Improving adherence to controller medication in children with asthma - the unexplored contribution of behavioural sciences

Strengths and Weaknesses/Forces et faiblesses:

Strong focus on vulnerable children, with some consideration to this in the sampling strategy (e.g. quota sampling by SES quintile, which is an ecological approach that could be balanced with an individual participant self-report by parents). The researchers should collect data on SES and other vulnerability factors, and not rely solely on the ecological deprivation measure. Sampling strategy seems convenience based and more effort could be done to ensure a more representative sample of patients.

Could more clearly delineate in a table what is collected from parents and/or children (when old enough), but appreciate analytic plan to examine and consider these data when collected from both; could consider looking at level of agreement as a predictor variable in your analyses.

I would have liked to see the aspect of vulnerable children status more strong considered in the analysis plan. Is a binary unmet social need as vulnerable or not the best (why not dose response)? Not sure how feasible it will be to look at referrals as explanatory variable in Aim 2 if you are making referrals for all who will accept them (what are the counts expected for this?).

The recognized approach for mediation could have been explained in the proposal (and not just cited). Power analysis includes Aim 1 and Aim 2, but does not discuss Aim 3; some of the estimates/a priori values are weakly justified (some preliminary work could help identify parameters for power calculations).

KT plan is somewhat traditional without clear goals and target audiences, and no discussion of how findings may inform future intervention is discussed in this section, especially given the evocation of potential impact/significance of the work, including in LMIC.

There is still some outstanding feedback from the previous reviews that seem unaddressed in terms of addressing developmental trajectories of child/child’s age in the analysis plan.

**Review Type / Type d'évaluation:**	Reviewer 3 / Évaluateur 3
**Name of Applicant / Nom du chercheur:**	Drouin, Olivier
**Application No. / Numéro de demande:**	461392
**Agency / Agence:**	CIHR/IRSC
**Competition / Concours:**	Project Grant/Subvention Projet
**Committee / Comité:**	Psychosocial, Sociocultural & Behavioural Determinants of Health 2/Déterminants psychosociaux, socioculturels et comportementaux de la santé 2
**Title / Titre:**	Improving adherence to controller medication in children with asthma - the unexplored contribution of behavioural sciences

Budget Recommendation/Recommandation budgétaire:

None

Please indicate your appraisal of the integration of sex as a biological variable as a strength, weakness, or not applicable to the proposal./Prière de sélectionner une option pour donner votre évaluation de l'intégration du sexe comme variable biologique en tant que point fort ou point faible de la proposition, ou en tant qu'élément non applicable à la proposition.

- [x] **Strength/Point fort**
- [ ] **Weakness/Point faible**
- [ ] **Not applicable/Non applicable**

Please indicate your appraisal of the integration of gender as a socio-cultural determinant of health as a strength, weakness, or not applicable to the proposal./Prière de sélectionner une option pour donner votre évaluation de l'intégration du genre comme déterminant socioculturel de la santé en tant que point fort ou point faible de la proposition, ou en tant qu'élément non applicable à la proposition.

- [x] **Strength/Point fort**
- [ ] **Weakness/Point faible**
- [ ] **Not applicable/Non applicable**

**Review Type / Type d'évaluation:**	Reviewer 3 / Évaluateur 3
**Name of Applicant / Nom du chercheur:**	Drouin, Olivier
**Application No. / Numéro de demande:**	461392
**Agency / Agence:**	CIHR/IRSC
**Competition / Concours:**	Project Grant/Subvention Projet
**Committee / Comité:**	Psychosocial, Sociocultural & Behavioural Determinants of Health 2/Déterminants psychosociaux, socioculturels et comportementaux de la santé 2
**Title / Titre:**	Improving adherence to controller medication in children with asthma - the unexplored contribution of behavioural sciences

Sex and/or Gender Considerations/Notions de sexe et/ou de genre:

Consider aspects of both sex (of child) and gender (of parent), using validated gender measures. Will explore potential for stratified analyses, instead of ensuring power for these.

Review Type/Type d'évaluation: SO Notes /Notes de l'agent scientifique
Name of Applicant/Nom du chercheur: Drouin, Olivier
Application No./Numéro de demande: 461392
Agency/Agence: CIHR/IRSC
Competition/Concours: 2021-04-01 Project Grant/Subvention Projet
Committee/Comité: Psychosocial, Sociocultural & Behavioural Determinants of Health 2/Déterminants psychosociaux, socioculturels et comportementaux de la santé 2
Title/Titre: Improving adherence to controller medication in children with asthma - the unexplored contribution of behavioural sciences

---

Assessment/Évaluation:

Strengths (including SGBA considerations):

The reviewers recognized the importance of childhood asthma and adherence to the medications prescribed to manage it. They recognized, too, the potential benefits of the information the proposed research will contribute. The sophisticated way in which the application addressed the roles played by social inequalities was especially impressive.

Strengths identified during the discussion include: (a) Additional information will be available from a complementary dataset, which adds to the potential information yield of the project and (b) Sex and gender issues were addressed well.

Weaknesses (including SGBA considerations):

The reviewers identified some weaknesses. The investigators are referred to their detailed comments for the specifics.

Budget:

No issues.

****

Note: The final rating of the application, provided in the Notice of Recommendation (NOR) and Notice of Decision (NOD), is the averaged rating of the peer review committee members following the discussion of

**Review Type/Type d'évaluation:**	SO Notes /Notes de l'agent scientifique
**Name of Applicant/Nom du chercheur:**	Drouin, Olivier
**Application No./Numéro de demande:**	461392
**Agency/Agence:**	CIHR/IRSC
**Competition/Concours:**	2021-04-01 Project Grant/Subvention Projet
**Committee/Comité:**	Psychosocial, Sociocultural & Behavioural Determinants of Health 2/Déterminants psychosociaux, socioculturels et comportementaux de la santé 2
**Title/Titre:**	Improving adherence to controller medication in children with asthma - the unexplored contribution of behavioural sciences

Assessment/Évaluation:

the application during the committee meeting, and therefore may differ from the ratings provided by the assigned reviewers in their respective reviews.

Remarque : La cote définitive de la demande, qui apparaît dans l'avis de recommandation et l'avis de décision, représente la moyenne des cotes accordées par les membres du comité d'évaluation par les pairs après avoir débattu de la demande à la réunion du comité. Elle peut donc différer de celle donnée par les évaluateurs dans leur évaluation respective.

``````````````````````````````````````````````````````````````````````````````````````````````````````
``````````````````````````````````````````````````````````````````````````````````````````````````````
